# A Novel Human-Like Collagen Hydrogel Scaffold with Porous Structure and Sponge-Like Properties

**DOI:** 10.3390/polym9120638

**Published:** 2017-12-13

**Authors:** Xi Song, Chenhui Zhu, Daidi Fan, Yu Mi, Xian Li, Rong Zhan Fu, Zhiguang Duan, Ya Wang, Rui Rui Feng

**Affiliations:** 1 Shaanxi Key Laboratory of Degradable Biomedical Materials, School of Chemical Engineering, Northwest University, 229 North Taibai Road, Xi’an 710069, China; songxi93@126.com (X.S.); zch@nwu.edu.cn (C.Z.); li_xian1214@163.com (X.L.); rongzhanfu@nwu.edu.cn (R.Z.F.); duan11170@163.com (Z.D.); lanmao1998@163.com (Y.W.); alice2017innu@163.com (R.R.F.); 2 Shaanxi R&D Center of Biomaterials and Fermentation Engineering, School of Chemical Engineering, Northwest University, 229 North Taibai Road, Xi’an 710069, China

**Keywords:** human-like collagen, sponge-like, porous hydrogel, cartilage tissue engineering, biological cross-linker

## Abstract

The aim of this research was to prepare a novel sponge-like porous hydrogel scaffold based on human-like collagen (HLC) that could be applied in cartilage tissue regeneration. In this study, bovine serum albumin (BSA) was used as a porogen to prepare the porous hydrogel, which had not been previously reported. Glutamine transaminase (TGase) was used as the cross-linker of the hydrogel, because it could catalyze the cross-linking of BSA. During the crosslinking process, BSA and HLC were mixed together, which affected the cross-linking of HLC. When the cross-linking was completed, the non-crosslinked section formed pores. The microstructure, porosity, swelling properties, and compressive properties of the hydrogel were studied. The results showed that the pore size of the hydrogel was between 100 and 300 μm, the porosity reached up to 93.43%, and the hydrogel had rapid water absorption and suitable mechanical properties. Finally, we applied the hydrogel to cartilage tissue engineering through in vitro and in vivo research. The in vitro cell experiments suggested that the hydrogel could promote the proliferation and adhesion of chondrocytes, and in vivo transplantation of the hydrogel could enhance the repair of cartilage. In general, the hydrogel is promising as a tissue engineering scaffold for cartilage.

## 1. Introduction

Articular cartilage has a special organization that has low cellularity, and avascular and alymphatic characteristics, meaning the regeneration and self-repair ability of articular cartilage is poor [1,2,3]. Articular cartilage injury by trauma or degenerative pathology can lead to joint pain, osteoarthritis (OA), and even cause joint dysfunction and disability [4,5]. Traditional techniques for cartilage repair include marrow stimulation technique (microfracture), osteochondral autograft implantation, and allograft transplantation. Although these methods have a certain restorative effect, each has its limitations [6]. The marrow stimulation technique is not lasting, osteochondral autograft implantation technology lacks regenerative transparent cartilage tissue, and allograft transplantation tends to cause immune rejection [7,8]. To address these issues, engineering scaffolds have been attracting attention in the field of repair and regeneration of cartilage defects.

The ideal tissue engineering scaffold should have a porous interconnected structure, good biocompatibility, biodegradability, and workability [9,10]. The tissue engineering scaffolds should have a porous interconnected structure and high porosity, necessary to provide sufficient space for growth, attachment, and proliferation of cells and secretion of extracellular matrix [10]. Some researchers considered that the porosity of cartilage scaffold should be more than 90%, and the best pore size is about 200 µm [11,12,13]. Conventional techniques for producing porous tissue engineering scaffolds include solvent casting and particulate leaching, phase separation and emulsion freeze drying, and gas foaming and electrospinning [14,15,16]. Solvent casting and particulate leaching, phase separation and emulsion freeze drying, and gas foaming easily form a non-interconnected pore structure and a nonporous skin layer at the scaffold surface. In addition, these techniques may use toxic organic solvents that can adversely affect the biocompatibility of the scaffold [17,18,19]. The scaffold formed by electrospinning has the advantages of high porosity, good connectivity, and uniformity of pores, while electrospinning is slow and uses organic solvents [20]. In addition to these techniques, advance manufacturing techniques, such as the Fused deposition technique and the three-dimensional (3D) printing technique, have been used to accurately prepare porous scaffolds, according to the presupposed shape and structure [14,21]. The main issue with the fused deposition technique is that it is not suitable for materials that are not resistant to high temperatures [22]. The 3D printing technique is complicated and costly, so the use of this technology is limited [23]. Each technique has disadvantages and limitations.

To prepare an excellent cartilage scaffold, we researched a novel porous hydrogel scaffold based on human-like collagen (HLC), which was prepared by a convenient and rapid method. This preparation method, without using any organic solvents, is safe and non-toxic, and the porous hydrogel prepared by this method has a homogeneous and highly-connected porous structure. Unlike conventional hydrogels [24,25], the porous hydrogel with sponge-like properties can quickly squeeze and absorb water in a wet state. In addition, being close to the natural extracellular matrix (ECM), the porous hydrogel, having excellent biocompatibility, is conducive to cell adhesion, migration, and differentiation, promotes the transport of nutrients and waste, and provides sufficient space and mechanical stability for tissue formation. Therefore, the porous hydrogel is an ideal cartilage tissue engineering scaffold material.

HLC is a novel genetically engineered protein created with recombinant Escherichia *coli* BL21 highly expressed cDNA, from reverse transcription from human mRNA. Its product was isolated and purified, and human-like collagen was obtained [26]. In addition to the advantages of collagen, HLC has excellent water solubility, low immunogenicity, good product stability, and is virus-free [26,27]. Due to these properties, HLC is considered a promising biomaterial. HLC’s ability to be applied in all aspects of biomedical engineering have been researched, including as a soft tissue filler [28], a hemostatic sponge [29,30], and a vascular scaffold [31]. 

In this study, we prepared a sponge-like 3D porous hydrogel based on HLC by using efficient and non-toxic Glutamine transaminase (TGase) as a cross-linking agent. Bovine serum albumin (BSA) and sodium chloride (NaCl) were used as porogens of the hydrogel. The TGase can catalyze the cross-linking of HLC to form a stable structure of the hydrogel, but it cannot catalyze the BSA cross-linking, so the addition of the BSA affects the cross-linking degree of the hydrogel. NaCl can also affect the cross-linking of the hydrogel. As a result, with the combined action of both, the hydrogel has a highly-connected 3D porous structure and has sponge-like properties. The method for making pores has never been reported. The physical and chemical properties of the hydrogel were analyzed by measuring the internal structure, density, porosity, swelling performance, and mechanical properties of the hydrogel. Then, we attempted to use the hydrogel as a cartilage scaffold, to evaluate the biocompatibility and cartilage repair capacity of the hydrogel through cytology and zoological experiments. The results showed that the hydrogel would have a huge application potential in the field of cartilage tissue engineering. The HLC^c^ hydrogel, without BSA, was used as the control group.

## 2. Materials and Methods 

### 2.1. Materials

Human-like collagen (HLC, China patent number: ZL01106757.8, *M*_r_ = 97,000) was supplied by our laboratory. Bovine serum albumin (BSA) was obtained from Amresco (Solon, OH, USA). Glutamine transaminase (TGase) was purchased from Shanghai Yuanye Biotechnology Co., Ltd. (Shanghai, China). The Dulbecco’s Modified Eagle’s Medium (DMEM)/high glucose (Hyclone), fetal bovine serum (FBS, BI), and pancreatin (Hyclone) were purchased from Xian Maoda Biotechnology Co., Ltd. (Xi’an, China). The Cell Counting Kit-8 (CCK-8) and the Live & Dead viability assay kit were provided by KeyGEN Biological Technology Development Ltd. Co. (KGA317, Nanjing, China). All other chemicals were of analytical grade and were used without further purification.

### 2.2. Synthesis of HLC^S^ and HLC^C^ Hydrogels

The preparation of the HLC^S^ hydrogel is was follows. First, HLC (120 mg/mL) and BSA (80 mg/mL) were completely dissolved in ultrapure water at 37 °C. NaCl (35 mg/mL) was added into the solution. After mixing thoroughly, the TGase (6 U/mL) was added and completely dissolved in the solution by stirring. Then, the mixture was cross-linked at 4 °C for 10 h to obtain the HLC^S^ hydrogel. Thereafter, the hydrogel was washed with ultrapure water for 3 days to remove free BSA, TGase and NaCl molecules. Finally, the hydrogels were frozen at −80 °C for 4 h followed by lyophilization in a freeze drier for 48 h.

The HLC^C^ hydrogel was prepared using the same method but without BSA.

### 2.3. SDS-PAGE of the Water Extract of HLC^S^ Hydrogel

The HLC^S^ hydrogel was immersed in ultrapure water for 24 h to provide the water extract. The water extract was diluted to the appropriate concentration. Then, HLC solution (0.1%), BSA solution (0.1%), and TGase solution (0.1%) were prepared. After the preparation of the sample, sodium dodecyl sulfate polyacrylamide gel electrophoresis (SDS-PAGE) was performed using the Laemmli method, then the gel being removed and stained with Coomassie Blue R-250 (Amresco, Solon, OH, USA) for about 30 min. Finally, we observed the experimental results after decolorization of the gel.

### 2.4. Morphology of Hydrogels

The surface morphology of the hydrogel was observed by a scanning electron microscope (SEM, Carl Zeiss, Oberkochen, Germany) at an accelerating voltage of 15 kV. 

### 2.5. Density and Porosity of Hydrogels

The following formula was used to calculate the density (*ρ*) of the hydrogels:

*ρ* = (4*M*)/(*πD*^2^*h*) (1)

where *M* is the mass, *D* is the diameter, and *h* is the height of the hydrogel.

The porosity of hydrogels was calculated using the following equation:

*Porosity* (%) = (*W*_2_ − *W*_3_ − *W*_s_)/(*W*_1_ − *W*_3_) × 100% (2)

where *W*_1_ is the quality of the 10 mL centrifuge tube filled with absolute ethanol, *W*_2_ is the total quality of the 10 mL centrifuge tube with hydrogel after immersing the hydrogel in absolute ethanol, *W*_3_ is the remaining mass of absolute ethanol and the 10 mL centrifuge tube after removal of the hydrogel, and *W*_s_ is the dry weight of the hydrogel.

### 2.6. Swelling Behavior of Hydrogels

The swelling behavior of the hydrogel was examined in phosphate buffered solution (PBS, pH 7.4) at 37 °C. The freeze-dried hydrogel was weighed and recorded (*M*_0_). The hydrogel was immersed in the PBS (pH 7.4) and removed at a predetermined point in time. After removing the surface solution of the hydrogel with filter paper, it was weighed and recorded (*M*_1_). The swelling ratio of the hydrogel was defined with the following equation:

*Swelling ratio* (%) = (*M*_1_ − *M*_0_)/*M*_0_ × 100%. (3)

### 2.7. Compressive Mechanical Properties of Hydrogels

The compression properties of the wet hydrogel (15 mm in diameter and 10 mm height) were tested using an INSTRON 5565 Materials Testing Machine (Instron, Norwood, MA, USA) at a loading rate of 10 mm/min. The compression strain of the wet HLC^C^ hydrogel and the wet HLC^S^ hydrogel were 60% and 80%, respectively.

### 2.8. Cell Culture and Viability Analysis

The hydrogel sterilized with cobalt (Co^60^) irradiation was immersed in the DMEM/high glucose culture medium (0.1 g/mL) at 37 °C for 72 h (GB/T16886). Rat articular chondrocytes were seeded on 96-well plates at a density of 10^4^ cells/well. After incubation for 24 h in a 37 °C incubator containing 5% carbon dioxide (CO_2_), the hydrogel extracts were added to the 96-well plates (100 μL/well). The cytotoxicity of the hydrogel extracts was determined using the CCK-8 assay after 1, 3, 5 and 7 days of culture. The relative cell growth (%) was calculated as:

*Relative cell growth* (%) = (*OD*_test_ − *OD*_blank_)/(*OD*_control_ − *OD*_blank_) × 100%. (4)

The mean value of eight parallel samples was determined, and the whole test was repeated three times.

### 2.9. Cell Attachment and Proliferation Analysis

The sterilized hydrogel was soaked in the DMEM/high glucose culture medium. Then, the saturated hydrogel was placed in a 24-well plate. A 100 μL of cell suspension containing 10^5^ cells was dropped onto each hydrogel sample. After 3 h of culture, 1 mL of fresh DMEM medium was added to each well. The cell-adhered hydrogel was cultured in incubators for 1, 3, 5 and 7 days, and the medium was changed daily. The viability of chondrocytes cultured on hydrogels was evaluated by Live & Dead cell viability assay. The live cells/dead cells were observed and imaged using by a fluorescence microscope (Olympus IX-51, Tokyo, Japan).

After 7 days of chondrocytes seeding, the morphology of the cell-adhered hydrogel was observed by SEM (Carl Zeiss, Oberkochen, Germany). The cell-adhered hydrogels were fixed with 2.5% glutaraldehyde for 24 h at 4 °C. Then, the hydrogels were gradually dehydrated with gradient ethanol solution (30%, 50%, 70%, 90%, 95%, 100%), and each gradient was dehydrated for 15 min and dried. Finally, the cell-adhered hydrogel sections were observed by SEM after gold coating.

### 2.10. Animal Implantation and Histological Evaluation

All animals were obtained from the Xi’an Jiaotong University (Xi’an, China). The study was conducted in accordance with relevant national legislation on the use of animals for research and the protocol was approved by the Animal Ethics Committee of Northwest University (NWU201705153). This study used healthy New Zealand white rabbits weighing 3–4 kg. Under general anesthesia, a cartilage defect (4.0 mm in diameter, 4.0 mm in depth) was created in the trochlear groove of the left leg using a dental grinding machine (Saeshin, STRONG 102, Daegu, Korea). The rabbits were randomly divided into three groups: the HLC^C^ group, the HLC^S^ group, and the control group (defect only). The rabbits were euthanized 12 weeks after the surgery. 

The harvested cartilage specimens were fixed in 10% formalin for 7 days and then decalcified in 15% ethylenediaminetetraacetate dihydrate (EDTA) solution for 30 days. After being embedded in paraffin, the samples were cut into 5 μm sections. Then, the sections were stained with hematoxylin and eosin (H&E) and Safranin O-fast green to detect the morphology and glycosaminoglycan (GAG) distribution. The histological staining was observed and imaged under an optical microscope connected to a charged-coupled device (CCD) camera (DP25, Olympus, Tokyo, Japan).

### 2.11. Statistical Analysis

All experiments were conducted at least three times unless otherwise stated. All the data were expressed as mean ± standard deviation and analyzed by one-way analysis of variance (ANOVA) using Origin 8.5 software (OriginLab, Northampton, MA, USA). *p* < 0.05 was considered statistically significant. 

## 3. Results and Discussion

### 3.1. Preparation of the HLC^S^ Hydrogel

**Figure 1:**
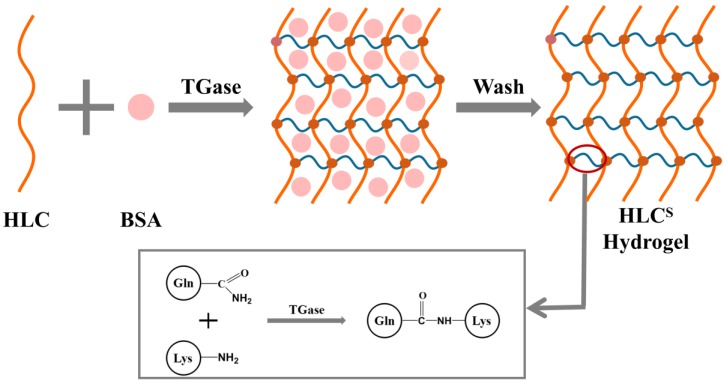
The cross-linking mechanism and schematic chemical structure of the HLC^S^ (human-like collagen) hydrogel.

The cross-linking mechanism and schematic chemical structure of the HLC^S^ hydrogel is shown in Figure 1, formed by the reaction of the γ-carboxamide group of a glutamine residue and the ε-amino group of a lysine residue in HLC. The TGase can catalyze the cross-linking reaction of two groups of HLC to form a relatively protease-resistant intermolecular or intramolecular ε-(γ-glutamyl) lysine isopeptide bond, thus forming a stable 3D network structure [32]. However, the TGase cannot catalyze the cross-linking reaction of BSA. During the crosslinking process, the HLC molecules around the BSA molecules were not completely cross-linked, which affected the cross-linking degree of the hydrogel. Therefore, after the completion of cross-linking, BSA only was physically mixed in the 3D network structure of the hydrogel, and those non-crosslinked section formed pores. Meanwhile, the addition of sodium chloride (NaCl) also affected the cross-linking of the hydrogel by changing the ionic strength of the solution. Changes of the ionic strength in solution could affect protein solubility. At low temperatures (the crosslinking temperature was 4 °C), suitable ion concentrations of NaCl could lead to a certain degree of phase separation [33,34,35]. In this state, HLC was crosslinked by TGase, so the HLC^S^ hydrogel formed a sponge-like 3D porous structure. In the end, the BSA, NaCl, and TGase molecules were washed out.

**Figure 2:**
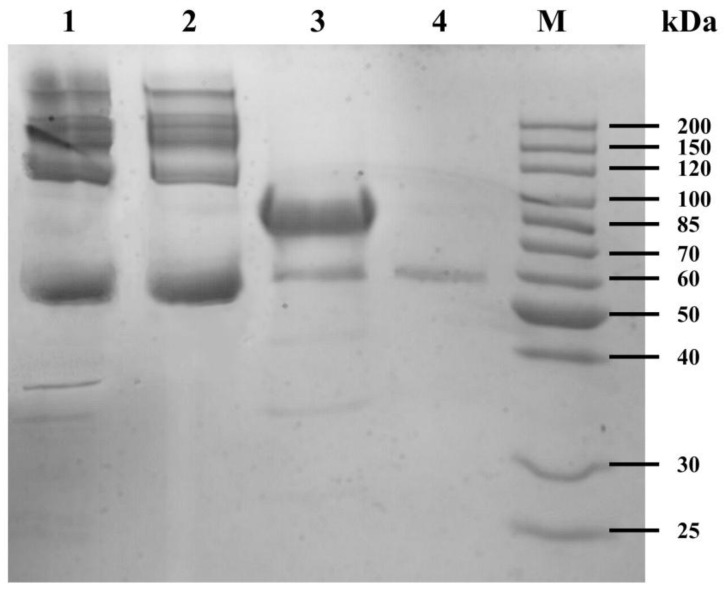
Sodium dodecyl sulfate polyacrylamide gel electrophoresis (SDS-PAGE) of the water extract of HLC^S^ hydrogel. Lane 1 is the water extract of HLC^S^ hydrogel. Lanes 2, 3, and 4 are bovine serum albumin (BSA) solution, HLC solution and TGase solution, respectively.

As seen in Figure 2, the electrophoretic band of the water extract of HLC^S^ hydrogel was like the electrophoretic band of the BSA solution, where there was no electrophoretic band of the HLC solution. This result showed that BSA did not participate in the cross-linking reaction and could be washed out of the HLC^S^ hydrogel. The electrophoretic bands of the TGase solution were partially overlapped with the electrophoretic bands of the water extract of the HLC^S^ hydrogel. However, as a catalyst, the enzyme was not directly involved in the reaction, so the TGase was also washed out by ultrapure water.

**Figure 3:**
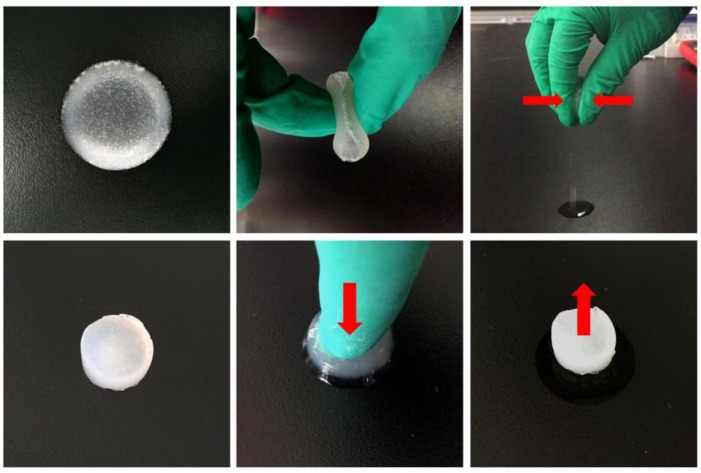
The appearance and water absorption properties of the wet HLC^S^ hydrogel.

Figure 3 shows the appearance and water absorption properties of the wet HLC^S^ hydrogel. The wet HLC^S^ hydrogel is translucent and has excellent flexibility and water absorption capacity. In addition, the hydrogel can also be formed into different shapes with a mold, which satisfies the requirements for clinical transplantation.

### 3.2. Morphology, Density, and Porosity of Hydrogels

**Figure 4:**
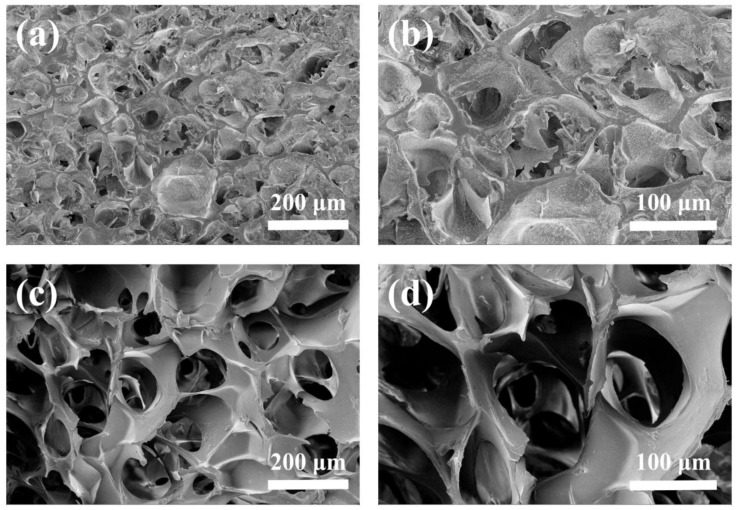
SEM images of hydrogels: (**a**) HLC^C^ hydrogel, Mag = 100×; (**b**) HLC^C^ hydrogel, Mag = 200×; (**c**) HLC^S^ hydrogel, Mag = 100×; (**d**) HLC^S^ hydrogel, Mag = 200×.

The SEM image of HLC^C^ hydrogel and HLC^S^ hydrogel are shown in Figure 4. Both hydrogels have a porous structure. However, the HLC^C^ hydrogel pore connectivity is poor, and the pore size is less than that of the HLC^S^ hydrogel. The HLC^C^ hydrogel mainly formed pores by vacuum freeze drying and the addition of NaCl. However, the presence of BSA affected the cross-linking of HLC, thus the HLC^S^ hydrogel has a highly-connected porous structure. Furthermore, the HLC^S^ hydrogel exhibited a relatively homogeneous pore structure, while the HLC^C^ hydrogel showed a non-homogeneous porous structure. Moreover, from Figure 4, the pore size of the HLC^S^ hydrogel was in the range of 100 to 300 μm. In general, a closed and non-homogeneous pore structure is not conducive to the proliferation and uniform distribution of cells, so the structure of the HLC^S^ hydrogel was more conducive to cell adhesion and proliferation.

**Figure 5:**
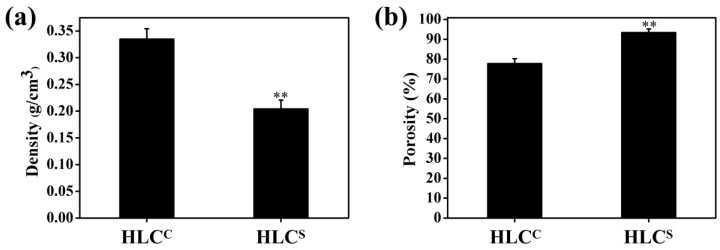
(**a**) Density of hydrogels; (**b**) Porosity of hydrogels (**p < 0.01, HLC^S^ hydrogel compared with HLC^C^ hydrogel).

The densities of the HLC^C^ and HLC^S^ hydrogels were 0.33 and 0.20 g/cm^3^, respectively (Figure 5a). The porosities of the HLC^C^ and HLC^S^ hydrogels were 77.76% and 93.43%, respectively (Figure 5b). The porosity of the HLC^S^ hydrogel was significantly higher than that of the HLC^C^ hydrogel, while the density was significantly lower than the HLC^C^ hydrogel. This is consistent with the SEM results due to the higher degree of cross-linking of the HLC^C^ hydrogel. As an ideal tissue engineering hydrogel, a porous interconnected structure and high porosity are required, which are important for nutrition supply and cell migration [36]. In brief, the HLC^S^ hydrogel has a highly-connected porous structure and an excellent porosity of more than 90%, and a pore size in the range of 100 to 300 μm, all of which make HLC^S^ hydrogel better for the repair and regeneration of cartilage tissue [11,12,13].

### 3.3. Swelling Ratio of Hydrogels

**Figure 6:**
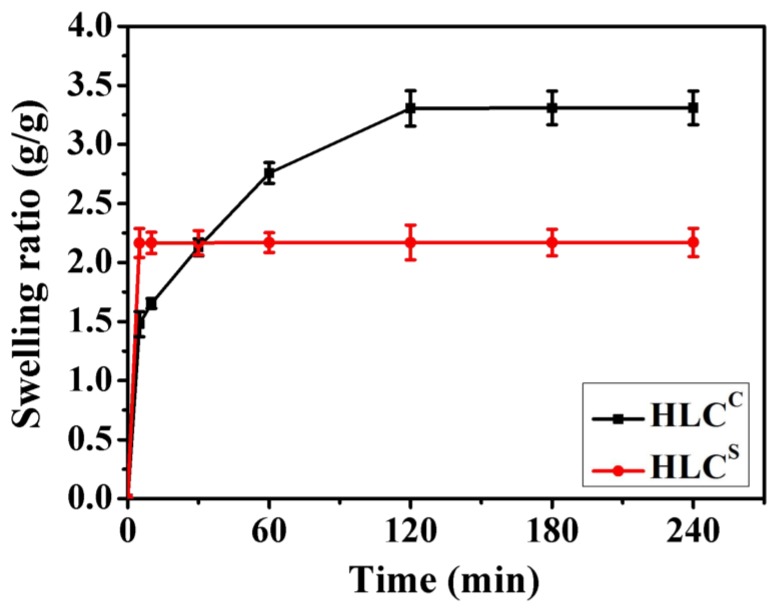
Swelling ratio of hydrogels in phosphate buffered solution (PBS) (pH 7.4).

As a biomedical material, the swelling ratio of the hydrogel is an important element in assessing the efficacy of the material. The swelling ratio represents the water absorption capacity of the hydrogel. In this study, the swelling ratios of the hydrogels in PBS (pH 7.4) were detected, as shown in Figure 6. The HLC^S^ hydrogel exhibited excellent water absorption capacity, achieving swelling equilibrium at about 5 min, while the HLC^C^ hydrogel took 120 min to achieve swelling equilibrium. The highly-connected porous structure of the HLC^S^ hydrogel allows the hydrogel to quickly absorb water, demonstrating the sponge-like properties of the HLC^S^ hydrogel. The swelling ratio of the HLC^C^ hydrogel was higher than the HLC^S^ hydrogel, likely due to the influence of the high cross-linking density [37]. HLC is a hydrophilic macromolecule, containing many hydrophilic groups that can attract water molecules. The HLC^C^ hydrogel has a high cross-linking density, smaller pore size, and poor pore connectivity, so the HLC^C^ hydrogel can retain more moisture and therefore have a higher swelling ratio after swelling equilibrium. However, the HLC^S^ hydrogel has a low cross-linking density, large pore size, and good pore connectivity. Although the hydrophilic groups on the HLC^C^ can attract water molecules but the water is easily lost from the pores, so the swelling ratio of HLC^S^ hydrogel is lower than that of the HLC^C^ hydrogel. In short, due to its excellent water absorption capacity, the HLC^S^ hydrogel can quickly absorb joint fluid and fill damaged tissue as a cartilage scaffold.

### 3.4. Compressive Mechanical Properties of Hydrogels

**Figure 7:**
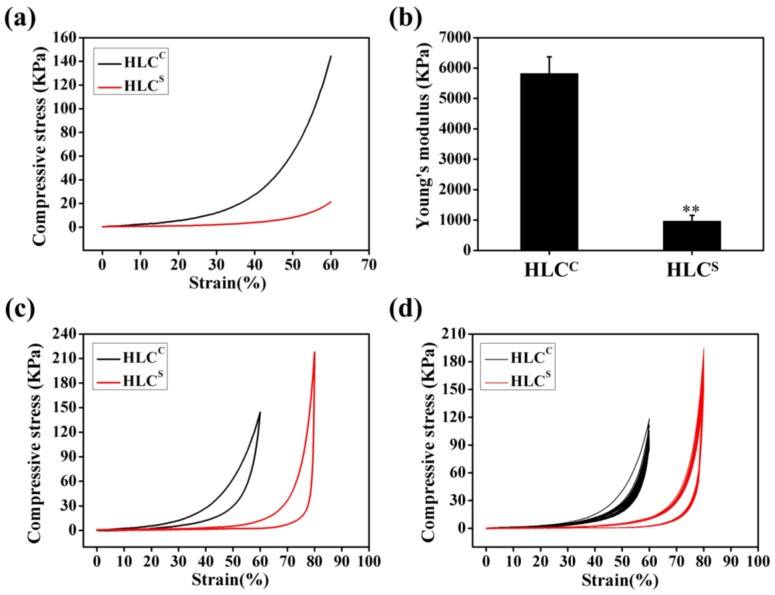
Compressive mechanical properties of hydrogels in the wet state: (**a**) Compressive stress-strain curves of the hydrogels (ε = 60%); (**b**) Young’s modulus of the hydrogels (ε = 60%); (**c**) Compressive stress-strain curves of the hydrogels under one compressing and releasing cycle (ε_HLC_^c^ = 60% and ε_HLC_^s^ = 80%); (**d**) 10 cycles of compressive fatigue curves of hydrogels (ε_HLC_^c^ = 60% and ε_HLC_^s^ = 80%).

The mechanical properties are one of the most important indicators that the properties of the hydrogel can be matched to the tissue specificity of the extracellular matrix (ECM) [38]. Compression is the primary means of mechanical testing of hydrogel materials. The compressive stress-strain curve of the HLC^C^ hydrogel and the HLC^S^ hydrogel (ε = 60%) in the wet state is shown in Figure 7a. The Young’s modulus of the HLC^C^ hydrogel and the HLC^S^ hydrogel were 5819.06 ± 552.55 KPa and 960.31 ± 193.63 KPa, respectively (Figure 7b). The Young’s modulus of the HLC^C^ hydrogel was significantly higher than that of the HLC^S^ hydrogel, due to the high cross-linking density of the HLC^C^ hydrogel. High levels of cross-linking form more isopeptide bonds, which stabilizes the HLC^C^ skeleton, resulting in the increase of the modulus. In the field of tissue engineering, cartilage tissue requires scaffolds with higher strength mechanical properties. 

The compressive modulus of the natural articular cartilage was reported between 0.1 and 2 MPa by Naseri et al. The compressive modulus values of native cartilage obtained from different sources and test conditions are different [39]. However, scaffolds with good mechanical properties are not conducive to retaining a high porosity [10]. Good mechanical properties provide mechanical support for new tissues, while high porosity provides a suitable 3D environment for chondrocytes growth and nutrient transport [40,41,42]. A balance between mechanical properties and porous structure is key to the success of any scaffold. Obviously, HLC^C^ hydrogel has better mechanical properties but poor pore connectivity and low porosity, which are not suitable for attachment and proliferation of chondrocytes. Conversely, the HLC^S^ hydrogel not only has excellent pore connectivity and high porosity, but also has mechanical properties that sufficiently meet the needs of cartilage scaffolds. Therefore, the HLC^S^ hydrogel has considerable potential as a cartilage tissue engineering scaffold.

Figure 7c shows that the HLC^C^ hydrogel could support a 60% compressive strain, whereas the HLC^S^ hydrogel could support a compressive strain of 80%. These two hydrogels would return to their initial shape after the release of the compressive force. The cyclic compression curve of the hydrogels is a non-linear closed curve. When the hydrogel absorbs water, the swelling rate of the hydrogel is less than the release rate of the machine, which might be the cause of the shape of this curve. The 10 cycles of compression fatigue curves for the HLC^C^ hydrogel (ε = 60%) and the HLC^S^ hydrogel (ε = 80%) are shown in Figure 7d. After a compression cycle, the hydrogels underwent some plastic deformation. The HLC^S^ hydrogel changed little, but the HLC^C^ hydrogel significantly changed. This might be due to sponge-like structure of the HLC^S^ hydrogel that exhibited an excellent elastic property in the wet state.

### 3.5. Cell Viability Analysis

**Figure 8:**
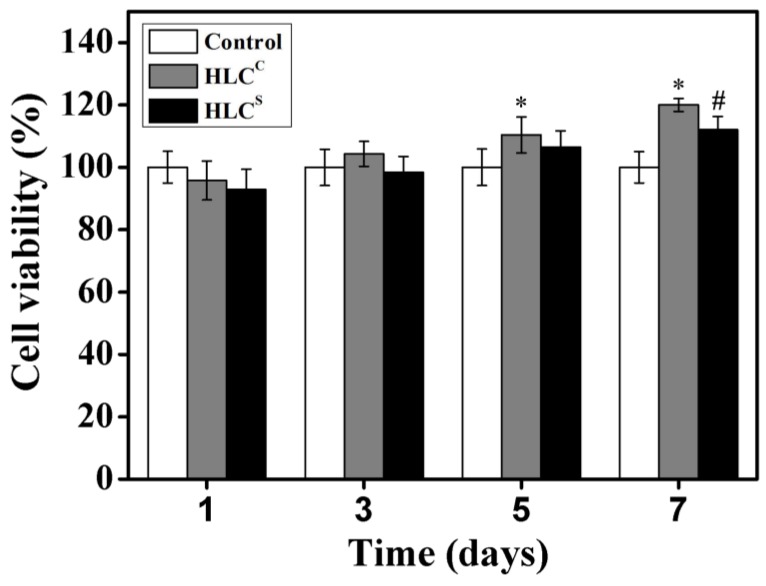
Cell viabilities of hydrogel extracts after culturing for 1, 3, 5, and 7 days (* represents the HLC^C^ hydrogel extracts compared with the control group, # represents the HLC^S^ hydrogel extracts compared with the control group).

Escherichia *coli* (*E. coli*) is a Gram-negative bacterium and HLC is highly expressed by recombinant Escherichia *coli* BL21, so proteins may also contain small amounts of endotoxin after being isolated and purified. Endotoxins are toxic to cells and can be leached from the hydrogel. Therefore, we examined the effect of the hydrogel extracts on cell proliferation using the CCK-8 assay to identify the cytotoxicity of the hydrogels. Cell viabilities of the chondrocytes in the extracts of the two hydrogels after culturing for 1, 3, 5 and 7 days are shown in Figure 8. After one day of culture, chondrocytes were not fully adapted to the new environment, so the cell viability was relatively low. In general, chondrocytes cultured with the extracts of two hydrogels showed proliferation at 3, 5, and 7 days. At 7 days, the chondrocytes were significantly proliferated (* *p* < 0.05, # *p* < 0.05), and both chondrocytes cultured with extracts of hydrogels had higher cell viability. Both extracts of hydrogels can promote the proliferation of chondrocytes. This may be due in part to HLC that is not completely cross-linked being leached out, promoting cell proliferation. After 7 days of culture, the cell viabilities of the HLC^C^ hydrogel and the HLC^S^ hydrogel were 119.99% and 112.1%, respectively. According to the ISO standards (ISO10993.12-2005), the toxicities of the HLC^C^ hydrogel and the HLC-S hydrogel were all grade 0. Simultaneously, we observed that HLC had an excellent effect on improving cell growth, which could promote the regeneration of damaged tissue. Therefore, the HLC^C^ and HLC^S^ hydrogels could promote cell growth and proliferation, which is in line with the requirements for tissue engineering scaffolds.

### 3.6. Adhesion and Proliferation of Chondrocytes on the Hydrogels

**Figure 9:**
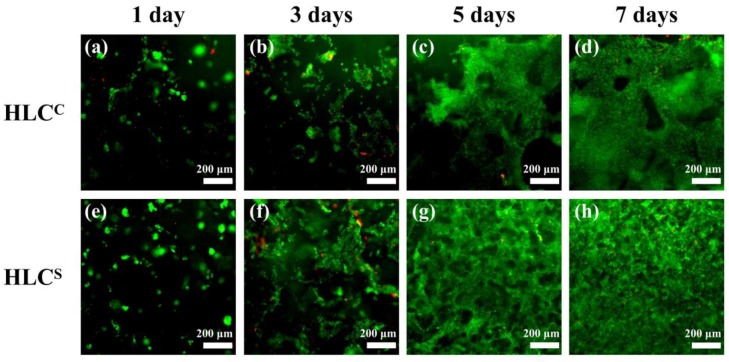
Fluorescence micrographs of cell morphology on the HLC^C^ hydrogel for (**a**) 1; (**b**) 3; (**c**) 5 and (**d**) 7 days. Chondrocytes seeded on the HLC^S^ hydrogel at (**e**) 1; (**f**) 3; (**g**) 5 and (**h**) 7 days. Live and dead cells were dyed green and red, respectively.

In this study, we observed the adhesion and proliferation of rat articular chondrocytes on the hydrogels by Live & Dead cell viability assay. The chondrocytes were cultured on the hydrogel for 1, 3, 5 and 7 days, and the cell adhesion is shown in Figure 9. The living cells (green) grew significantly more than dead cells (red), which indicates excellent cell viability of the hydrogels. By comparing the chondrocytes on the hydrogels at 1, 3, 5 and 7 days, we found that the chondrocytes on both hydrogels had significant proliferation. After 7 days of culture, the live cells on the hydrogel were tightly connected and almost no dead cells were present (Figure 9d,h). As shown in Figure 9c,d,g,h, the chondrocytes on the HLC^C^ hydrogel only grew on the hydrogel surface, while the HLC^S^ hydrogel image showed that many chondrocytes were distributed on the pore walls and the hydrogel surface. In addition, many chondrocytes were found in the depths of the HLC^S^ hydrogel. Because the focal lengths of the different planes were different, some chondrocytes that adhered to the HLC^S^ hydrogel are slightly blurred in Figure 9. The fluorescent staining results also showed that the HLC^S^ hydrogel had a 3D porous structure and the cell distribution of the HLC^S^ hydrogel performed more uniformly.

**Figure 10:**
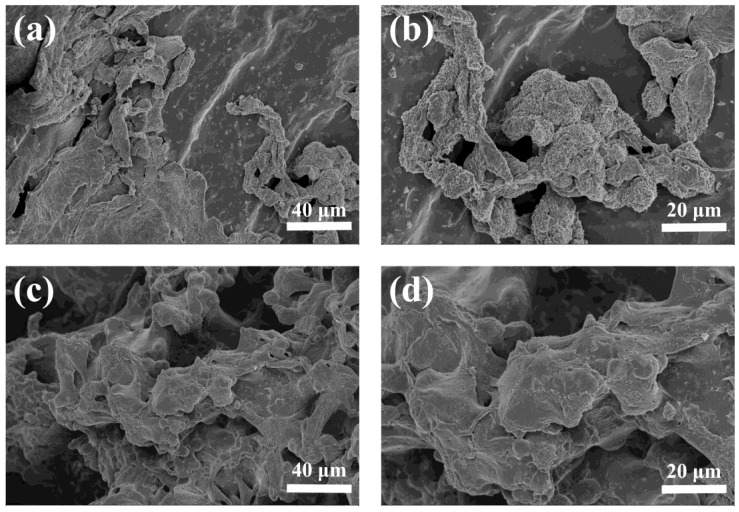
The SEM micrographs of the chondrocytes adhering to the hydrogels: (**a**) Chondrocytes seeded on the HLC^C^ hydrogel, Mag = 500×; (**b**) Chondrocytes seeded on the HLC^C^ hydrogel, Mag = 1000×; (**c**) Chondrocytes seeded on the HLC^S^ hydrogel, Mag = 500×; (**d**) Chondrocytes seeded on the HLC^S^ hydrogel, Mag = 1000×.

The adhesion of chondrocytes to hydrogels was also evaluated by SEM. The morphology of the chondrocytes adhering to the hydrogels after 7 days in culture is shown in Figure 10. Chondrocytes adhered and proliferated on both hydrogels and exhibited good morphology. The shape of the chondrocytes attached to the surface of the hydrogels was spindle or irregular. The chondrocytes on the HLC^S^ hydrogel adhered to the pore walls and were uniformity distributed (Figure 10c,d), whereas the chondrocytes on the HLC^C^ hydrogel only adhered to the surface of the hydrogel and were unevenly distributed (Figure 10a,b). This was consistent with the results of the Live and Dead cell viability assay (Figure 9). In conclusion, the fluorescence micrographs and the SEM micrographs of the chondrocytes both suggested that the HLC^C^ and HLC^S^ hydrogels possessed excellent biocompatibility. Moreover, the 3D porous structure of HLC^S^ hydrogel is more conducive to the adhesion and proliferation of chondrocytes.

### 3.7. Observation and Histological Evaluation of Cartilage Repair

**Figure 11:**
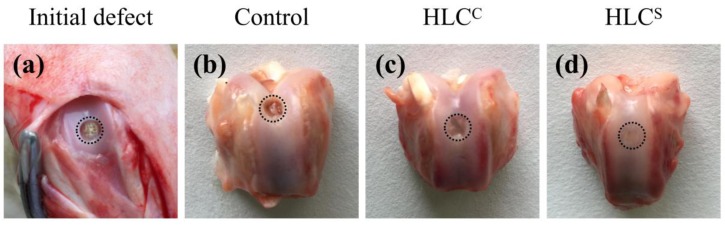
Macroscopic images of the cartilage joint: (**a**) The initial defect; (**b**) The control groups 12 weeks after surgery; (**c**) The HLC^C^ groups 12 weeks after surgery; (**d**) The HLC^S^ groups 12 weeks after surgery.

The repair status of the cartilage defects 12 weeks after surgery is shown in Figure 11. Compared to the picture of initial defect in Figure 11a, the defect in the control group was not filled (Figure 11b), whereas the defects were observed to be filled by a semitransparent tissue in the HLC^C^ group (Figure 11c) and the HLC^S^ group (Figure 11d). The defect in the HLC^C^ group was shallower and was filled with semitransparent cartilage-like tissue, but remained discontinuous. The defect in the HLC^S^ group was filled with a uniform cartilage-like tissue with a flat surface. Only a blurred boundary was observed between the defect and the adjacent normal cartilage.

**Figure 12:**
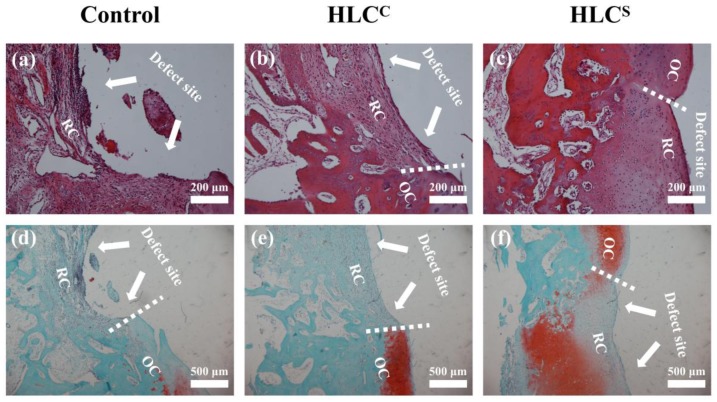
Histological analysis of the cartilage defect area: (**a**) The control group 12 weeks after surgery, stained with H&E; (**b**) The HLC^C^ group 12 weeks after surgery, stained with H&E; (**c**) The HLC^S^ group 12 weeks after surgery, stained with H&E; (**d**) The control group 12 weeks after surgery, stained with Safranin O-fast green; (**e**) The HLC^C^ group 12 weeks after surgery, stained with Safranin O-fast green; (**f**) The HLC^S^ group 12 weeks after surgery, stained with Safranin O-fast green. The defects are marked with white arrows in the images. OC: original cartilage tissue. RC: repaired cartilage tissue.

The microscopic morphology of the cartilage defect area was observed by H&E staining and Safranin O-fast green staining 12 weeks after surgery (Figure 12). The defect in the control group without implant was not filled, and only a few loose fibrous tissues were found (Figure 12a,d). The Safranin O-fast green staining image also indicated that the defect in the control group was not filled with no GAG deposition (no red). The defect in the HLC^C^ group was partly filled with relatively dense fibrous tissue, but the fibrous tissue was thinner than the adjacent normal cartilage tissue (Figure 12b,e). Like the control group, the defect in the HLC^C^ group was not observed with Safranin O-fast green staining (red), without GAG deposition. Compared to the control group and the HLC^C^ group, the defect in the HLC^S^ group was filled with uniform cartilage-like tissue, which was well-integrated with adjacent normal cartilage tissue (Figure 12c,f). In addition, regenerated cartilage tissue in the defect site was observed to be stained with Safranin O-fast green staining (red), indicating that there was adequate GAG deposition in the regenerated cartilage tissue. In summary, the gross observation and histological analysis of the regenerated tissues revealed that the implant of the HLC^S^ group could effectively repair articular cartilage defects.

## 4. Conclusions

In this study, we used TGase as a cross-linker to prepare the HLC^S^ hydrogel for cartilage tissue engineering. We proposed a novel pore-forming method that used BSA as porogen to obtain the porous structure of the hydrogel. From the SEM images, the HLC^S^ hydrogel had a highly-connected 3D porous structure. The physical and chemical properties of the hydrogel were explored by a series of experiments. The results showed that the HLC^S^ hydrogel had high porosity, rapid water absorption capacity, and suitable mechanical properties. In vitro cell culture results demonstrated that chondrocytes could adhere to the HLC^S^ hydrogel and had good cell viability and cell morphology. The in vivo experiments demonstrated that the HLC^S^ hydrogel could effectively repair rabbit articular cartilage damage. Therefore, the HLC^S^ hydrogel could be an ideal biomaterial for cartilage tissue engineering.
