# Erratum: A Novel Human-Like Collagen Hydrogel Scaffold with Porous Structure and Sponge-Like Properties. *Polymers*, 2017, *9*, 638

**DOI:** 10.3390/polym10030304

**Published:** 2018-03-12

**Authors:** Xi Song, Chenhui Zhu, Daidi Fan, Yu Mi, Xian Li, Rong Zhan Fu, Zhiguang Duan, Ya Wang, Rui Rui Feng

**Affiliations:** 1 Shaanxi Key Laboratory of Degradable Biomedical Materials, School of Chemical Engineering, Northwest University, 229 North Taibai Road, Xi’an 710069, China; songxi93@126.com (X.S.); zch@nwu.edu.cn (C.Z.); li_xian1214@163.com (X.L.); rongzhanfu@nwu.edu.cn (R.Z.F.); duan11170@163.com (Z.D.); lanmao1998@163.com (Y.W.); alice2017innu@163.com (R.R.F.); 2 Shaanxi R&D Center of Biomaterials and Fermentation Engineering, School of Chemical Engineering, Northwest University, 229 North Taibai Road, Xi’an 710069, China

The authors wish to make a change to the published paper [1]. In Section 2.2, “HLC (120 g/mL) and BSA (80 g/mL)” has been changed to “HLC (120 mg/mL) and BSA (80 mg/mL)”. The mistake was due to the authors’ oversight. To avoid misleading readers, we would like to update the data in the article. The authors apologize for any inconvenience this may have caused.

The manuscript will be updated and the original will remain online on the article webpage http://www.mdpi.com/2073-4360/9/12/638.
